# Metabolism Controls the Balance of Th17/T-Regulatory Cells

**DOI:** 10.3389/fimmu.2017.01632

**Published:** 2017-11-27

**Authors:** Licheng Sun, Jinrong Fu, Yufeng Zhou

**Affiliations:** ^1^Children’s Hospital and Institute of Biomedical Sciences, Fudan University, Shanghai, China; ^2^Key Laboratory of Neonatal Diseases, Ministry of Health, Shanghai, China

**Keywords:** metabolism, Th17 cell, T-regulatory cell, immunoregulation, cell polarization

## Abstract

Accumulating evidence indicates that metabolism reprogramming is critically important to T cell differentiation, and manipulating metabolic pathways in T cells can shape their fate and function. During T cell differentiation, metabolism provides T cells with energy as well as precursors for various biological processes. Some key metabolic reactions, such as glycolysis, oxidative phosphorylation and fatty acid oxidation, are also considered to play important roles in T cell activation and differentiation. In this review, we will explain why cellular metabolism is important for the Th17/T-regulatory (Treg) cell balance and how metabolism reprogramming impacts this balance. Moreover, we will also discuss some important metabolic sensors, such as mammalian target of rapamycin, AMP-activated protein kinase, and some nuclear receptors. In addition, we will review specific small molecular compounds, which can shift the Th17/Treg cell balance and, therefore, have promising therapeutic roles. Finally, potential methods of manipulating Th17 cell metabolism for treating Th17-associated diseases will be discussed.

## Introduction

Naïve T cells are quiescent and largely metabolically inactive. After stimulation, T cells start to proliferate and differentiate (1). During this process, metabolism reprogramming is necessary and crucial and can meet energy requirements and provide various substrates that are indispensable for T cell proliferation and differentiation. Furthermore, increasingly more reports have indicated that, as well as acting as an energy source, metabolic processes can also be regulators of T cell differentiation (2, 3).

Naïve CD4^+^ T cells can differentiate to various T helper cells (Th), including Th1, Th2, Th9, Th17, and T-regulatory (Treg) cells. These T helper cells have diverse functions and act as a pivotal part in both mediating adaptive immunity and autoimmune diseases (4, 5). According to their special cytokines and function, CD4^+^ T helper cells were first classified as two subsets, Th1 and Th2. The Th1/Th2 theory was established 20 years ago and reported that dysfunction in the immune system and autoimmune diseases were induced by the unbalance of Th1 and Th2 (6, 7). This hypothesis was not substantially challenged until the discovery of Th17. Th17 cells were first recognized as novel CD4^+^ T helper cells in 2005; they predominately secrete interleukin-17 (IL-17) and protect the host against pathogens (8, 9). On the other hand, excess Th17 cells can induce autoimmune disease and inflammation (9). Treg cells are also a lineage of CD4^+^ T cells. The function of Treg cells is to maintain immune homeostasis and limit the excessive immune response. Although the function of Treg and Th17 are completely different, Th17 and Treg share many important elements during differentiation (10, 11).

The unique relationship between Th17 and Treg has been discussed for a long time, and many molecular mechanisms that control the balance of Th17 and Treg have been revealed. Despite the molecular regulating network, accumulating evidence shows that processes involving metabolism are also important in Th17 and Treg differentiation. Therefore, here, we will focus on how metabolism processes influence the balance of Th17 and Treg. Initially, some general ideas about Th17 and cellular metabolism will be discussed, and then we will provide an overview of recent investigations about how metabolic reprogramming regulates the balance of Th17/Treg. Finally, potential therapeutic options will be briefly discussed.

## Overview of Th17 And Treg

Th17 cells were identified as a new lineage of CD4^+^ T helper cells in 2005, although the signature cytokine Th17 was described earlier (12). In fact, the path leading to discovery of Th17 cells began in 2003 with the finding that experimental autoimmune encephalomyelitis (EAE) in the murine model was caused by high levels of IL-23 rather than IL-12 and Th1 cells (13). Consequently, researchers found that the function of IL-23 is to promote differentiation and proliferation of the IL-17-secreting cells, which were eventually identified as Th17. Nevertheless, IL-23 alone is unable to make naïve T cells differentiate into Th17 cells (9, 14, 15). Subsequently, many research teams have found that polarization of Th17 can be efficiently induced by transforming growth factor-β1 (TGF-β1) and IL-6, which activate Smad family proteins and STAT3, respectively (16, 17). STAT3 further promotes and maintains the expression of Th17-specific genes (1, 18). The master transcription factors of Th17 are retinoic acid-related orphan receptors-γt (RORγt), which can directly regulate the expression of Th17-specific genes, such as *CCR6, CD161, IL17a, Il17f*, and *Il23r*. On the other hand, RORγt is also a critical component in a larger network of transcription factors like Stat3, IRF4, or BATF (1, 18, 19). In addition to the ability to fight against extracellular microbial organisms and mediate autoimmune disease, Th17 cells are also involved in allograft rejection. Antagonism of IL-17 can promote the survival rate in a rat cardiac allograft model; furthermore, in a lung transplantation model, expression of IL-17 and IL-23 was also demonstrated to be upregulated at the site of allograft rejection (20, 21).

It has been known for a long time that CD4^+^T cells can either activate or suppress immune responses. Treg is another lineage of CD4^+^T cells but it has a totally different function compared to Th17 and can modify the immune response, maintain immune tolerance to self, and prevent autoimmune disease (22). Generally, Treg can be classified as nTreg (natural Treg) and iTreg (induced Treg). Treg originating from the thymus is often named as nTreg, otherwise peripherally derived Treg is regarded as iTreg (22). The surface markers of Treg are CD4^+^ and CD25^+^, and its master transcription factor is Forkhead box P3 (Foxp3) (23). Foxp3 can provide Treg with suppressive activity and upregulate other Treg-specific genes, such as CD25, CTLA4, and IL-10. In mice, defective Foxp3 causes loss of immune tolerance and autoimmune disease. Moreover, CD4^+^, CD25^+^, and Foxp3^+^ are recognized as good markers for mouse Treg, although recently Foxp3 has also been found to be expressed in CD4^+^ and CD25^−^ cells (22). Notably, in humans, Foxp3 is not only expressed in Treg but also in some conventional T cells (22, 24). Like other T helper cells, Treg cells are differentiated from their naïve CD4^+^ T cells, but the main function of Treg is suppressing activation of the immune system (22). A lack of Treg causes autoimmune disease, and a high ratio of Treg/Th17 is associated with cancer incidence (23, 25).

Activation of T cell receptor (TCR) and costimulatory signaling is needed to initialize the differentiation of all CD4^+^ T helper cells. However, after activation, different lineages of specific cytokines drive the differentiation of distinct cell subsets. TGF-β1 is required for differentiation of both Th17 and peripheral iTreg (26). This common point suggests that differentiation of these two subsets is related. When the TGF-β1 signaling pathway is activated, the expression of RORγt and Foxp3 is upregulated. Whether naïve T cells polarize to a Th17 phenotype or a regulatory phenotype largely depends on the surrounding microenvironments (27, 28). IL-6 has been recognized as a major cytokine that drives the differentiation of Th17 cells. Foxp3 can inhibit Th17 development by directly binding to RORγt. Without IL-6, the TGF-β signaling pathway reinforces this inhibition and favors the formation of Treg from naïve T cells. In the presence of IL-6, STAT3 can be activated and Foxp3 is released from RORγt (29). During differentiation of Th17, the expression of IL-23R is also upregulated, and activated IL-23R can further induce Th17 differentiation. Moreover, activation of IL-23R is also very important for proliferation and maintenance of the phenotype of Th17 cells after differentiation. The IL-23 signaling pathway can also activate STAT3 and inhibit IL-10 production (19, 28, 30). It should also be noted that TGF-β1-induced Th17 differentiation can occur without IL-6, if there is sufficient IL-21. Some studies have shown that human naïve CD4^+^ T cells can be induced to Th17 by adding exogenous IL-21. Treating human naïve CD4^+^ T cells with IL-21 and TGF-β can upregulate the expression of both IL-23 and RORγt, while inhibiting the expression of Foxp3 (31). After differentiation, Th17 can secrete IL-21 and form an autocrine loop to further promote the Th17 phenotype. In summary, in the presence of TGF-β1, both IL-6 and IL-21 can induce differentiation of T cells to Th17. Otherwise, T cells will differentiate to Treg (32).

**Figure 1:**
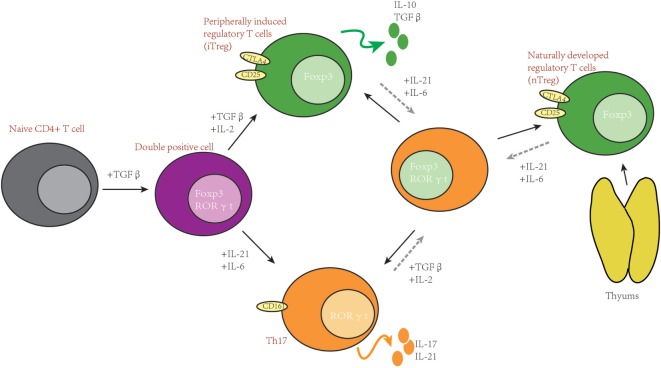
Th17 and T-regulatory (Treg) share important developmental elements. After stimulation, naïve T cells can be induced into RORγt and Forkhead box P3 (Foxp3) double-positive T cells. Furthermore, whether naïve T cells differentiate to Th17 or Treg depends on the surrounding cytokines. In some situations, Th17 and Treg can trans-differentiate.

Besides sharing a similar development pathway, in some conditions, Th17 and Treg can also trans-differentiate to each other (33–36). It has been reported that high concentrations of exogenous Th17-generating cytokines can convert Foxp3^+^ Treg cells into IL-17 secreting cells (35). On the other hand, it has also been reported that Treg can lose Foxp3 expression and go on to acquire an inflammatory function (36). Some investigators also indicate that there are intermediate cells during Th17 to Treg trans-differentiation, which are Foxp3 and IL-17 double-positive T cells (37) (please see Figure 1).

## Overview of Cellular Metabolism

### Glycolysis, Tricarboxylic Acid (TCA) Cycle, and Oxidative Phosphorylation (OXPHO)

Glycolysis refers to a sequence of cytosolic enzymatic reactions that convert glucose into pyruvate, generating energy. Generally, after being imported from the extracellular space by glucose transporter (Glut), glucose is initially converted to glucose 6-phosphate (by the rate-limiting enzyme hexokinase) and subsequently to fructose 6-P, spontaneously. A second rate-limiting enzyme, phosphofructokinase-1, enables the formation of fructose1, 6-diphosphate. After several steps, pyruvate is formed, which can then be processed in two ways. It is either imported to the mitochondria, with consequent conversion to acetyl-CoA. Acetyl-CoA can enter the TCA cycle to produce NADH and FADH2, which can generate ATP by OXPHO. One molecule of glucose metabolized by aerobic glycolysis can generate two molecules of ATPs. By contrast, if the products generated by glycolysis can be further metabolized by the TCA cycle and OXPHO, the theoretical maximum number of ATP molecules from one molecule of glucose can be generated: 32 ATPs in total. It should also be noted that glycolysis and the TCA cycle not only provide energy but also generate many intermediates for other metabolic pathways, such as the pentose phosphate pathway (PPP), hexosamine biosynthesis, amino acid biosynthesis, and *de novo* fatty acid synthesis (FAS). Another fate of pyruvate is conversion to lactate in the cytosol by lactate dehydrogenase (38). Pyruvate converted to lactate often occurs under hypoxic conditions. This process generates NAD^+^, which is consumed during glycolysis. When cells cannot regenerate NAD^+^ by OXPHO, the strategy of generating lactate can provide NAD^+^ and remove the limitation of glycolysis. Interestingly, with sufficient oxygen, sometimes cells still utilize glucose to generate lactate, which is known as aerobic glycolysis or the Warburg effect (3, 38, 39). This unique phenomenon has often been reported in tumor cells and, more recently, in T cells (40, 41). Moreover, it has been shown that aerobic glycolysis is crucial for polarization of T helper cells, especially Th17 (42, 43).

On the other hand, OXPHO is an important metabolic process for generating ATP molecules by means of the electron transport chain (ETC). NADH and FADH2 are catalyzed back into oxidized forms (NAD^+^ and FAD) by Complex I or II, respectively, in the mitochondria. The reactions can generate electrons and protons. Protons are transferred into the inter-membrane space of the mitochondria. At the same time, electrons are passed along the ETC from Complex III, first to cytochrome C and then to Complex IV, in which protons are finally transferred to molecular oxygen generating water (38, 44). Otherwise, differential electrical charges are created between the mitochondrial membranes during the process, and this electrical potential difference is utilized by ATP synthase, which can pump protons back to the mitochondrial matrix and generate ATP (38).

### Fatty Acid Metabolism

The fate of pyruvate generated by glycolysis is participation in *de novo* FAS. Pyruvate can convert into acetyl-CoA in the mitochondria. However, this acetyl-CoA must be transported back to the cytosol where FAS and cholesterol synthesis occur. Acetyl-CoA cannot be transported into the cytosol directly, it must condensate with oxaloacetate first to generate citrate and is then transported to the cytosol where the reverse reaction occurs, and citrate is re-converted to acetyl-CoA. Furthermore, the rate-limiting step in FAS takes place. Acetyl-CoA is carboxylated into malonyl-CoA by acetyl-CoA Carboxylase 1 (ACC1). Then, acetyl-CoA is converted into malonyl-CoA to generate longer acyl chains, the reaction of which is catalyzed by a multi-enzyme complex, FAS. Acyl chains can continue to elongate by adding acetyl molecules, but this is dependent on the availability of NADPH. The final product of FAS is palmitate, which can be further utilized to generate other fatty acids or lipoproteins (38, 45).

Fatty acid synthesis is an anabolic process that generates fatty acids to reserve energy. Conversely, fatty acid oxidation (FAO) can convert fatty acids to acetyl-CoA in order to generate energy for cell proliferation and activation. Initially, fatty acids can be transported into the mitochondria by carnitine palmitoyl transferase present on the mitochondrial membrane, specifically CPT1 and CPT2. Once transported into the mitochondria, long-chain fatty acids are metabolized by a series of enzymatic reactions (38, 46). At the same time, NADH, FADH2, and acetyl-CoA are generated. Furthermore, acetyl-CoA can feed into the TCA cycle for further oxidization, while NADH and FADH2 can be utilized as electron donors for OXPHO (46). As well as aerobic glycolysis, FAS and FAO are also important regulators for lymphocyte activation and differentiation, especially for Treg (45).

## Metabolic Reprogramming and Th17/Treg Balance

Naïve T cells are relatively quiescent cells and their TCR are not stimulated. Compared to activated T cells, naïve T cells are comparatively smaller in size and are arrested at the G0 stage; they rely on IL-7 for survival and maintain their quiescent state (47). Correspondingly, considering metabolic activity, naïve T cells require less energy because they do not divide and secrete significant amounts of cytokines. Without the need to obtain the energy rapidly, naïve T cells usually utilize nutrients in the most effective way to generate maximum ATP through complete oxidization. In this way, OXPHO to yield ATPs is the main energy source for naïve T cells. For glucose, they are usually degraded by glycolysis and then feed into the TCA cycle for further oxidization, which ensures utilizing glucose most efficiently and generating 32 ATPs from one molecule of glucose.

However, after stimulation, CD4^+^ naïve T cells become highly proliferative and differentiate into T helper cells (Th). Without doubt, T cell activation and differentiation require metabolic reprogramming to support their rapid expansion and bio-function (48), and a series of changes in metabolic pathways occurs. First, the transporters for nutrients are highly expressed, such as glucose transporter (Glut) for transporting glucose and Alanine–Serine–Cysteine Transporter (ASCT2) for transporting glutamine (43, 49). Then glycolysis, OXPHOS, PPP, hexosamine pathway, and fatty acid metabolism all become active. Each aspect of the orchestration is important, and subtle differences in these metabolic programs will lead to differentiation of naïve CD4^+^ T cells to different T helper cells lineages (48, 50–52). The PPP can generate ribose-5-phosphate (R5P) and NADPH. R5P is used as a substrate to synthesize nucleotides, and NADH is an important mediator in many metabolism reactions. The hexosamine pathway generates UDP-*N*-acetyl glucosamine, which can be used for posttranslational modifications of proteins (53, 54).

T cell activation needs metabolic reprogramming, and different metabolic profiles can impact the Th17/Treg balance. One research group investigated 400 metabolites by high-resolution non-targeted Q exactive–mass spectrometry, and liquid chromatography/gas chromatography–MS (LC/GC–MS) demonstrated that Th17 and Treg have different metabolic profiles. Compared to Treg, Th17 cells have high levels of pyruvate, lactate, and PPP intermediates, while Treg cells have more TCA-cycle intermediates, which suggests Th17 cells rely more on aerobic glycolysis and glutamine oxidation and Tregs rely more on pyruvate oxidation (43). Examination of the level of amino acids in Th17 and Treg subsets, no clear subtype patterns can be observed. Notably, in both Th17 and Treg, the level of aspartate is much higher than other amino acids (about 10-fold higher), which may indicate that aspartate has an important function in Th17 and Treg (55). Examination of Acyl-carnitines indicates that Treg cells have higher levels of C2 and C4-OH carnitine compared to Th17. These metabolites represent acetyl and β-hydroxybutryl CoA species, respectively. The MS data also show a drop in oleyl-carnitine (C18:1). This fall in a long-chain acyl-carnitine coupled with the rise in C4-OH (hydroxybutyryl carnitine) is consistent with the fact that the level of FAO is high in Treg, and supports the idea that compared to other T helper cells Treg cells use fatty acids as a fuel to generate energy (43). Gene expression data show similar results. Gene arrays demonstrate that glucose metabolism-related genes are highly expressed in Th17. However, the fatty acid transporter CPT1A and ETC component cytochrome c is highly expressed in Treg compared with Th17. Interestingly Th17 and Tregs preferentially expressed a single isoform of each glycolytic enzyme, with utilization of Glut as glucose transporters. In Tregs, only Glut1 is expressed, and in Th17 both Glut1 and Glut3 are expressed, with Glut1 being dominantly expressed. As discussed above, hexokinase is the rate-limiting enzyme in glycolysis. Hk1 is preferentially expressed in Treg, while HK2 and HK3 is preferentially expressed in Th17 (43).

Glycolysis is especially important for the development of Th17. Research has shown that when naïve T cells are stimulated, the PI3K signaling pathway will be activated. Furthermore, while increasing the expression of Glut, PI3K signaling pathway can promote Glut trafficking to the cell membrane (56). Without Glut, the development of T helper cells is impaired, while Tregs are less affected (51). As discussed above, combined with other reports, it can be concluded that glycolysis is much more active in Th17 compared to Treg (43, 57); defective glycolysis can impair Th17 differentiation dramatically. By blocking glycolysis, Th17 cell proliferation and cytokine production are inhibited (58, 59). One reason concerns energy; to some extent, Treg cells are more like naïve T cells and need relatively less energy. Moreover, in order to provide energy for expansion and exerting function, other T helper cells, including Th17, need an abundant amount of ATP in a very short time. Therefore, Th17 cells need to rely on glycolysis for their development. Nevertheless, unlike naïve T cells, pyruvate does not feed into the TCA cycle and it is alternatively converted to lactate. In this process, each molecule of glucose can generate two molecules of ATPs (60). Despite the low usage rate for energy production, glycolysis is still independent of differentiation of T helper cells, especially Th17. This may be due to the fact that glycolysis can generate energy much faster (61).

Another hypothesis is that glycolysis is crucial for initializing metabolic programs and can also generate many metabolic intermediates for other metabolic pathways, such as the synthesis of proteins, nucleic acids, and lipids in the process of development of T helper cells (48, 62, 39). However, this hypothesis is controversial. Some reports have argued that these biosynthetic pathways are only slightly upregulated (less than 10%) by aerobic glycolysis (63). In the past, many investigations were focused on how metabolites impact the synthesis of proteins, nucleic acids, and lipids. Recent reports, however, provide some new ideas. The metabolic pathways may influence Th17 or Tregs in other ways. One report demonstrated that UDP-GlcNAc, a sugar-nucleotide, can affect the balance of Th17 and Treg; UDP-GlcNAc promotes *N*-acetyl glucosamine branching of Asn (N)-linked glycans. In addition, N-glycan branching can inhibit endocytosis of IL2Rα. Furthermore, this process drives naïve T cell differentiation to Treg. Interestingly, the biosynthesis of UDP-GlcNAc acts *via* the hexosamine pathway. In Th17 cells, increasing glycolysis and glutaminolysis compete for fructose-6-phosphate and glutamine in the hexosamine pathway, and then lower N-glycan branching. Indeed, GFPT1 is specifically downregulated by Th17 cells, and kifunensine, which blocks GlcNAc branching, can skew the Treg to Th17 (54). Another report has shown that metabolites can impact the epigenetic mechanism, further influencing the Th17 and Treg balance. Compared to Treg, a metabolite named 2-hydroxyglutarate (2-HG) accumulates in Th17. It was also revealed that (aminooxy)acetic acid(AOA) can switch Th17 re-differentiation to Tregs by inhibiting the activity of glutamate-oxaloacetate transaminase (GOT1), which is a transaminase and mediate glutamine metabolism (55). In normal conditions, GOT1 can convert aspartate to oxaloacetic acid, while generating electrons for ETC. However, upon ETC inhibition, it reverses to generate aspartate in the cytosol and rescue cell survival and proliferation (64). However, the reason that AOA can promote Foxp3 expression and inhibit Th17 differentiation is by generating a metabolite, 2-HG, rather than affecting Th17 proliferation (55). It was also reported that 2-HG is an inhibitor of TET1 and TET2; inhibition of TET1 and TET2 leads to hypermethylation of the Foxp3 gene locus and inhibition of Foxp3 transcription (55). Moreover, compared to manipulating glycolysis, AOA and kifunensine are aimed to the branch of the glucose metabolic pathway, which cause less side effects and have the potential to be developed as drugs to treat Th17-associated autoimmune diseases.

In addition to energy requirements and by-products, some reports have given other possible interpretations for the importance of glycolysis in T helper cells. As described above, GAPDH is an important enzyme engaged in glycolysis. When glycolysis is not active, in the absence of substrate, GAPDH will combine with the 3′ UTR of IFN-γ mRNA to disturb its translation. This provides a possible hypothesis that GAPDH enzymes involved in glycolysis (or other metabolic process) may modulate T cell differentiation. Moreover, it has been demonstrated that many metabolic enzymes can indeed transport to the nucleus. Furthermore, these enzymes can interact with mRNA to impact their stability or interfere with their translation (65). Aerobic glycolysis could keep GAPDH or other enzymes fully engaged and prevent their alternative function as inhibitors for T cell development (66). However, this hypothesis cannot explain how aerobic glycolysis can easily impact Th17 cells, apart from the evidence that enzymes involved in glycolysis can influence the expression of IL-17 or RORγt.

Although we have emphasized the importance of aerobic glycolysis, mitochondrial OXPHOS is still indispensable for T cell activation and proliferation. Inhibiting mitochondrial OXPHOS with oligomycin can completely abrogate the proliferation of TCR-activated T cell (66). Conversely, in the glucose-free medium, after TCR stimulation, T cells can still divide and express IL-2, which is regarded as an activation marker of TCR stimulation (44). NAD^+^ is an important component of ETC and also a co-factor of many biological reactions. A recently published paper shows that, in the presence of oligomycin, Jurkat cells can still proliferate by adding exogenous NAD^+^ to the medium. In this study, the authors also showed that failure to proliferate was due to the complete inability to synthesize aspartate without NAD^+^ rather than energy (64, 67). For Th17 and Tregs, NAD^+^ is reported to have the ability to specifically convert Treg into Th17 through the purinergic receptors, P2RX4 and P2RX7. Treating skin allograft mice *in vivo* with NAD^+^ can reduce the frequency of CD4^+^ CD25^+^ Foxp3^+^ Tregs and increase the frequency of Th17 cells. Interestingly, NAD^+^ can prolong allograft survival, which is paradoxical with the reducing of Tregs and increasing of Th17. The author attributes this to the fact that NAD^+^ can systematically increase CD4^+^ IL-10^+^ producing cells. After treatment with NAD^+^, around 50% of CD4^+^ T cells are IL-10-producing cells although Tregs are reduced (68). Sirtuins are a class of NAD-dependent deacetylases, and NAD^+^ is indispensable for the function of sirtuins. In Th17, SIRT1 can deacetylate RORγt and enhance Th17 cell generation (69). On the other hand, when mitochondrial OXPHOS occurs, mitochondrial complex III generates reactive oxygen species (ROS). Mitochondrial ROS (mtROS) can promote differentiation of Th17 cells. Knockout IEX-1 gene, which suppresses mtROS production can promote Th17 differentiation and aggravate arthritis in a mouse model. However, knockout IEX-1 gene does not impact Treg differentiation. It has also been reported that *N*-acetylcysteine, which is a specific inhibitor for mtROS production, can dramatically inhibit Th17 differentiation (70).

Lipid metabolism is also an indispensable element for the development of T helper cells. Lipids are the main components of cell membranes and provide energy and participate in cell signaling. Many recent reports have shown that lipid metabolism is an important regulator in T cell development (71). Both FAS and FAO are indispensable for T cell proliferation and differentiation (71–73). As described above, ACC1 is the rate-limiting enzyme for FAS. For Th17 cells, ACC1 is indispensable; Th17 cells depend on ACC1-mediated FAS to produce phospholipids for cellular membranes, while Treg is preferable to take up exogenous fatty acids. Impaired ACC1 function not only decreased FAS, but also decreased the level of glycolysis in Th17 cells, which can further skew Th17 differentiation to Treg (45). Moreover, ACC1 may be regarded as a potential target for clinical trials to treat Th17-related diseases. Soraphen A, which is a specific inhibitor of ACC1 can attenuate EAE in a mouse model (45). Another report also demonstrated that C75, which is an inhibitor of FAS, can reduce EAE disease (74). Madecassic acid has also been reported to ameliorate colitis in mice by downregulating the expression of ACC1 (75). On the other hand, it has also been reported that inhibition of ACC1 can decrease the level of FAS and increase the level of FAO (45, 76). Treg rely more on FAO to supply energy and, therefore, inhibiting ACC1 can also promote the development of Tregs (43, 77).

## Important Metabolic Sensors Controlling of Th17/Treg Balance

### Mammalian Target of Rapamycin (mTOR)

Mammalian target of rapamycin is a protein kinase of the phosphatidylinositol 3-kinase-related kinase family, which can sense the environment for cells and be activated by hormones, nutrients, and various stress conditions. mTOR contains two complexes, mTORC1 and mTORC2, which each have their own specific functions (78). A lack of mTORC1 signaling, in both human and murine naïve CD4^+^ T cells resulted in failure to differentiate to the Th17 lineages (79, 80). If both mTORC1 and mTORC2 are lost, naïve CD4^+^ T cells can only differentiate to Treg cells. Knockout of mTOR in mouse cells causes differentiation of naïve CD4^+^ T cells only to Treg cells, and expression of Foxp3 will be upregulated significantly (81). Consistent with these results, using rapamycin, an inhibitor of mTOR, can lead to a similar phenotype (81). Moreover, it has been shown that rapamycin can ameliorate EAE in a mouse model by means of the mTOR-STAT3 signaling pathway, which is important to Th17 development (82). In view of a molecular mechanism, some articles have reported that mTORC1 may regulate the development of Th17 cells by decreasing the expression of growth factor independent 1 transcriptional repressor (Gfi1) and increasing the expression of RORγt (79, 80). Inactivation of mTORC1 can cause Th17 cells to become more sensitive to the TGFβ signaling pathway, which can overcome STAT3 signaling further to influence the Th17 and Treg balance (81). In view of glycolysis, mTORC1 has the ability to upregulate the expression of Glut1 gene and promote glycolysis (83). In the early time of development of Th17, glutamine uptake increased significantly (43). Interestingly ASCT2, the transporter of glutamine, has been reported to be associated with the activation of mTORC1. ASCT2 is indispensable for mTORC1 activation in CD4^+^ T cells, and differentiation of Th17 is impaired in ASCT2-deficient mice. This finding partially explains how glutamine can modulate Th17 and Tregs development (49).

Conversely, the ability of mTOR to promote Th17 differentiation is through promoting the expression of hypoxia-inducible factor 1α (HIF1α). HIF1α is a transcriptional factor. Just like mTOR, it is also a well-known environmental sensor and metabolic regulator. Recently, increasingly more investigations have been focused on the ability of mTOR in T cell differentiation. Notably, both mTOR and HIF1α can promote glucose import and glycolysis at the transcriptional and translational level (84). Th17 cells have been shown to rely more heavily on glycolytic pathways than any other T cell subset (58, 79, 80). In the absence of HIF1α, the ability of murine naïve T cells to differentiate to Th17 cells are dramatically impaired (58). Some researchers have attributed this to the consequence of decreased glycolysis. It has been reported that HIF1α can promote glucose uptake and reinforce glycolysis by upregulating expression of Glut1 and pyruvate dehydrogenase kinase 1 (PDK1), respectively. Glut1 facilitates the transport of glucose across the plasma membranes of mammalian cells. On the other hand, PDK1 can prevent pyruvate feeding into the TCA cycle and drive it to be converted into lactate and then promote glycolysis. Conversely, expression of many glycolysis-related genes is downregulated in HIF1α mutation mice, such as Glut1, HK2, Ldha, and Pkm. All of these genes are rate-limiting enzymes (58, 85). At the transcriptional level, it has been reported that HIF1α can upregulate RORγt expression. Moreover, HIF1α can also form a complex with RORγt and p300 to further promote IL-17 expression. On the contrary, it has also been reported that HIF-1α attenuates Treg development by binding Foxp3 and targeting it for proteasomal degradation (58, 85).

Conversely, after TCR stimulation, naïve CD4^+^ T cells lacking mTOR tend to differentiate into Tregs, even in the absence of exogenous TGFβ. One research group found that proliferation of Treg is subtly linked to the transient inhibition of mTOR. Constant activation of mTOR can impair the function and proliferation of Treg *in vitro*. One possible explanation is that high mTOR activity may change the metabolic profile of Treg cells. As a consequence of the activation of mTOR, FA synthesis and aerobic glycolysis is activated, further inhibiting the development of Treg. However, the mechanism for these phenomena remains unclear (79, 86). These results still prove that levels of mTOR activity can influence the balance of Th17 and Treg.

### 5′ AMP-Activated Protein Kinase (AMPK)

**Figure 2:**
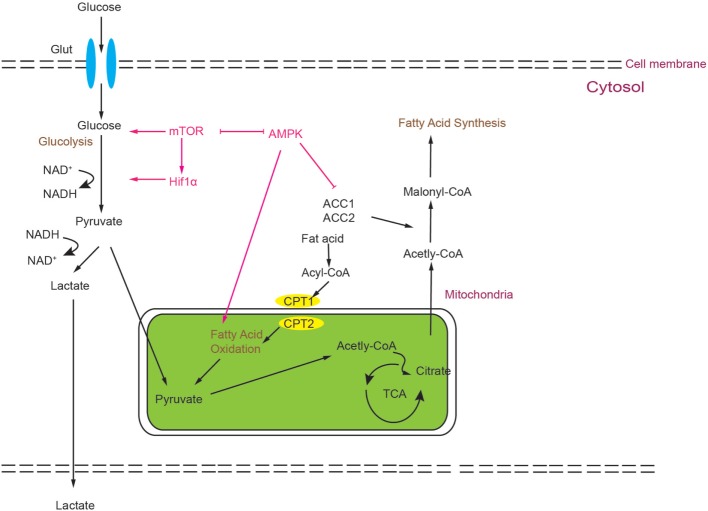
Simplified scheme of the metabolic processes activated during T cell development. Glucose is imported from the extracellular space by Glut1 and degraded by glycolysis to generate two pyruvate molecules, which can be converted to lactate or Acetyl-CoA. Acetyl-CoA can feed into the tricarboxylic acid (TCA) cycle. Alternatively, it can also be utilized as a precursor of FAS. Activation of mammalian target of rapamycin (mTOR) can modulate HIF1α activity, and both can promote glycolysis. mTOR and AMP-activated protein kinase (AMPK) act as negative regulators for each other. AMPK can inhibit glycolysis and promote fatty acid oxidation.

AMP-activated protein kinase is a heterotrimeric kinase complex, which is formed by α, β, and γ subunits. The γ subunit of AMPK contains a special domain, cystathionine-β-synthase that can sense the cellular AMP/ATP ratio and respond to the low energy conditions. In T cells, following TCR stimulation, AMPK is activated by the Ca2^+^-dependent protein. Once AMPK is activated, the energy-consuming metabolism is downregulated and the catabolic metabolism that can produce energy is upregulated. Unsurprisingly, AMPK can inhibit FAS and promote FAO (61, 87–89). Many important enzymes that are included in FAO and FAS can be regulated by the activation of AMPK, such as ACC1 and ACC2, almitoyl transferase I (CPT I), and sterol regulatory element-binding protein 1c (SREBP-1c). CPT I is a rate-limiting enzyme in FAO; otherwise, phosphorylated ACC1/2 and SREBP-1c can inhibit the synthesis of fatty acids and activate FAO (90). Another phenomenon is the relationship between AMPK and mTOR. AMPK and mTOR are negative regulators for each other, and AMPK can also negatively regulate the level of glycolysis (91). As described above, Th17 cells rely more on glycolysis, and Treg cells rely more on FAO, and the ability of AMPK to modulate metabolism indeed impacts the balance of Th17/Treg; AMPK is highly expressed and active in iTreg (92). According to this report, activated AMPK can drive naïve T cells to differentiate toward Treg cells both *in vitro* and *in vivo*. On the contrary, defective function in AMPK leads to an increase in mTOR activity and upregulation of glycolysis (90). Some studies have shown that treating naïve T cells with metformin, which is an activator of AMPK, can impair Th17 differentiation. Treating with metformin can increase lipid oxidation in T cells *in vitro*. *In vivo* administration of metformin was shown to be sufficient to decrease Glut1 expression (43, 77). Moreover, treating mice with metformin can also relieve several inflammatory diseases in mouse models (43, 77). In addition, treating mice with metformin can decrease the ratio of Th17:Treg in both colitis and asthma models, which can be attributed to the ability of decreasing the aerobic glycolysis and increasing the FAO (89, 93, 94). Notably, results from genetic and pharmacological approaches are not totally consistent. *In vitro* results compared to WT, AMPK^−/−^ CD4^+^ T cells have no more tendency to differentiate to Th17 cells. However, T cells from LKB1 knockout mice can produce more IL-17 than WT mice (95), which is upstream of AMPK and can activate 13 other kinases in the AMPK family (95, 96). The mechanism for this is still unclear and LKB1 may act through other pathways to impact Th17 differentiation (please see Figure 2).

### Nuclear Receptors (NRs) in the Th17/Treg Balance

Nuclear receptors are a class of transcriptional factors that can only regulate the expression of target genes when binding with the ligands. Exposure to the ligands leads to a conformational change, and NRs then acquire the ability to start transcription. Ligands of NRs are widely varied from hormones to metabolites (97). Now, many NRs are thought to be important metabolic regulators and they can participate in many metabolic processes during T cell development. These NRs can either promote or inhibit T cell activation and differentiation. Moreover, recent studies have shown that NRs play crucial roles in maintaining the Th17/Treg balances (21).

The aryl hydrocarbon receptor (AHR) is a conserved ligand NR in animals and, as a transcriptional factor, it is classified as being a member of the basic helix-loop-helix family. In the last decade, AHR was established for the ability to sense environmental toxins and protect hosts from being harmed. The external toxin, 2,3,7,8-tetrachlorodibenzo-p-dioxin (TCDD), and endogenous ligands, 6-formylindolo[3,2-b]carbazole (FICZ), are well-known ligands of AHR. However, recently the function of AHR in the development of T helper cells has been increasingly more described in research papers (98–100). AHR regulates Treg and Th17 cell differentiation in a ligand-specific manner. AHR activation by TCDD induced Treg cells that suppressed EAE, whereas AHR activation by FICZ interfered with Treg cell differentiation, boosted TH17 cell differentiation, and worsened EAE. But the detail mechanisms remain to be elucidated (99, 100). Some researchers have shown that AHR, together with HIF1α, can regulate the metabolic reprogramming during the development of Treg cells. In the early stage of Treg cells development, expression of metabolism-related genes is controlled by HIF1α, and at the later time points, AHR can promote HIF1α degradation to take over the control of these metabolic genes (101).

Estrogen-related receptor alpha (ERRα) is recognized as a key transcriptional factor in maintaining metabolic homeostasis and can regulate comprehensive genes involved in different metabolic pathways. These genes are related to glycolysis, mitochondrial energy metabolism, OXPHO, and fatty acid metabolism (102, 103). Interestingly, these important NRs are orphan receptors, and the endogenous ligands of ERRα have still not been found (104). It has been shown that in naïve T cells ERRα is weakly expressed. After TCR stimulation, expression of ERRα is dramatically upregulated (102, 103). XCT790, an antagonist of ERRα can decrease the level of energy metabolism in naïve T cells and impair the differentiation of all CD4^+^T cell lineages. It has been reported that the XCT790-induced reduction in T helper cells function is through upregulation of CPT1 levels, which can activate FAO and reduce glucose metabolism (102). This impairment could be rescued by adding exogenous fat acids. However, exogenous fat acids cannot restore T cell functions completely after differentiation, except for Treg cells (102). Genetic knockout ERRα shows similar results. In the mouse EAE model, the ERRα mutation shows restored symptoms compared to WT mice. Further analysis shows defective ERRα results in a decrease of Th17 polarization, but the differentiation and function of Treg cells are not affected. These results indicate that, compared to Treg, ERRα is more important for Th17 differentiation (102, 103).

The liver x receptors (LXRs) mainly regulate metabolism of cholesterol, fatty acids, and glucose homeostasis. The endogenous ligands of LXRs are recognized as oxysterols. LXRs impact the balance of Th17/Treg by regulating the genes involved in cholesterol metabolism. These genes include Abca1, Abcg1, and SREBP-1c (105). After binding, LXR can negatively regulate the expression of SREBP-1c and inhibit the cholesterol synthesis. These changes are disadvantageous to effector T helper cell development. LXR can also interact with AHR and inhibit its function (106, 107). *In vitro*, T0901317, which is an agonist of LXRs, can inhibit murine CD4^+^ naïve T cells differentiation to Th17 (106, 107), and inhibition of Th17 by LXRs can be overcome by adding mevalonate, which is a precursor of cholesterol. Moreover, LXR agonists also suppress expression of IL-17, RORγt, and other Th17-associated genes. In contrast, the antagonist of LXRs, GSK2033, enhances differentiation and proliferation of Th17 (106, 108). *In vivo*, activation of LXRs can relieve the symptoms of EAE in mouse models by reducing Th17 polarization (106).

Fortunately, most of the NRs involved in the Th17/Treg balance have their own specific inhibitors. Pharmacological modulators make it possible to manipulate the metabolic processes manually, providing an easy way to study the function of metabolic reprogramming in T cell development.

## Conclusion

As two important subsets of T helper cells, Th17 and Treg represent two arms of an immune response. Th17 cells can participate in the defense against extracellular bacterial and fungal infections. On the other hand, Treg cells can regulate the immune response and maintain immune homeostasis. Excessive activation of Th17 can lead to inflammation and autoimmune disease. Conversely, enhanced Treg function may cause tumorigenesis and can also be used by pathogens to escape the immune system. The balance of Th17 and Treg cells is critical for the health of the host. An appropriate balance between Th17 and Treg is a critical prerequisite for good health. Considering the close relationship between Th17 and Treg cells, and their plasticity, modulating the immunological balance between Th17 and Treg may represent a promising option for immune therapy.

As mentioned above, the field of immune metabolism has gained increasingly more attention in immunological research. Modulating cellular metabolism may be a feasible solution for adjusting the balance of Th17 and Treg. Both glycolysis and fatty acid can be regulated using small molecule inhibitors of key regulatory enzymes in these processes. Direct manipulation of cellular metabolism can shift the balance between Th17 and Treg cells (109).

**Figure 3:**
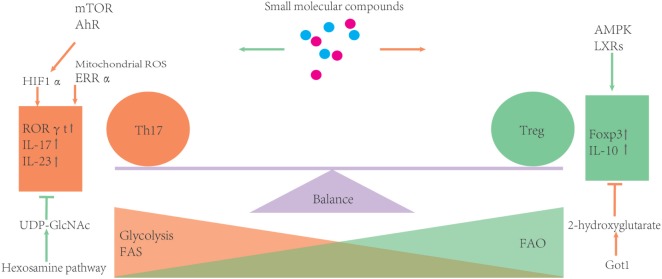
Metabolic processes can influence the balance of Th17/T-regulatory (Treg). During development, Treg rely more on fatty acid oxidation (FAO) as the energy source. Otherwise, glycolysis and FAS can promote Th17 differentiation. Several metabolic regulators or enzymes are involved in the balance of Th17 and Treg. Some small molecular compounds of these regulators or enzymes may provide an easy way to further investigate how certain metabolic routes impact the Th17/Treg balance.

Small molecular inhibitors or activators of metabolic enzymes are now becoming available, which makes it possible to manipulate metabolism of T cells much easier than ever before. Moreover, these small molecules can become potential drugs for clinical therapies. However, considering the complexity of metabolism, it is necessary to confirm findings derived from pharmacological inhibitors using animal disease models and clinical trials (please see Figure 3 and Table 1).

**Table 1:** Compounds and their effects on Th17 and T-regulatory (Treg) cells.

Compounds name	Functions	Effect on Th17 and Treg cells	Reference
Kifunensine	Blocking branching of GlcNAc and promoting the endocytosis of IL2Rα in Th17 cells	Promoting Th17 differentiation	(54)
(Aminooxy)acetic acid	An Inhibitor the of Got1; blocking the generation and accumulation of 2-hydroxyglutarate in cells; reducing the hypermethylation in the Forkhead box P3 gene locus	Skewing Th17 differentiate to Treg	(55)
*N*-acetylcysteine	A specific inhibitor for mitochondrial reactive oxygen species	Inhibiting Th17 differentiation	(70)
Soraphen A	A specific inhibitor of acetyl-CoA Carboxylase 1; decreasing the level of fatty acid synthesis (FAS) and glycolysis in Th17	Inhibiting Th17 differentiation; Attenuating experimental autoimmune encephalomyelitis (EAE) in animal model	(45)
C75	An inhibitor of FAS	Inhibiting Th17 differentiation	(74)
Rapamycin	An inhibitor of mammalian target of rapamycin; decreasing glucose uptaking and expression of HIF1α	Inhibiting Th17 differentiation; Attenuating EAE in animal model	(81–83)
Metformin	Agonists of AMP-activated protein kinase; decreasing aerobic glycolysis and increasing fatty acid oxidation	Decreasing the ratio of Th17:Treg in both colitis and asthma models	(77, 89, 93, 94)
FICZ	Endogenous ligands of AhR; increasing glycolysis; upregulating the expression of HIF1α	Promoting Th17 differentiation *in vitro*	(100)
CH-223191	An antagonist of AhR	Inhibiting Th17 differentiation *in vitro*	(101)
GSK2033	An inhibitor of LXR	Enhancing the differentiation and proliferation of Th17; attenuating EAE in animal model	(106, 108)

Metabolic factors driving Th17 development and those shaping the balance between Th17 and Treg have significant biological implications for the design and implementation of novel therapeutic interventions.

## Author Contributions

LS, the first author, contributed to collection of references and manuscript preparation. JF and YZ contributed to the editing of the manuscript.

## Conflict of Interest Statement

The authors declare that the research was conducted in the absence of any commercial or financial relationships that could be construed as a potential conflict of interest.
